# The pregnancy health and birth outcomes of women who underwent assisted reproductive technology: Results of a national survey 

**Published:** 2011

**Authors:** Lii-Shung Huang, Chi-Hwa Yen, Shu-Hsin Lee, Bih-Ching Shu, For-Wey Lung, Ching-Pyng Kuo, Wei-Ya Wu, Angel Yen-Chiao, Yen-Ju Lin, Hui-Sheng Lin, Ming-Chih Chou, Meng-Chih Lee

**Affiliations:** 1 School of Nursing, Chung Shan Medical University, Taichung, Taiwan.; 2 Institute of Medicine, Chung Shan Medical University, Taichung, Taiwan.; 3 Department of Family and Community Medicine, School of Medicine, Chung Shan Medical University and Hospital, Taichung, Taiwan.; 4 Department of Family Medicine, Taichung Hospital, Department of Health, Executive Yuan, Taichung, Taiwan.; 5 Department of Nursing, College of Medicine, Cheng Kung University, Tainan, Taiwan.; 6 Department of Medicine, Kaohsiung Armed Forces General Hospital, Kaohsiung, Taiwan.

**Keywords:** *Assisted reproduction technology* (*ART*), *Pregnancy health*, *Birth outcome*, *Taiwan Birth Cohort Study* (*TBCS*)

## Abstract

**Background:**

There is an upward trend for parents to resort to assisted reproductive technology (ART) treatment due to delayed childbirth or birth difficulties.

**Objective:**

This study investigates the pregnancy health and birth outcomes of women who underwent ART and analyzes the factors that influence birth weight to become<10 percentile when undergoing ART.

**Materials and Methods:**

This study analyzed results of the first wave of the Taiwan Birth Cohort study. Through stratified systematic sampling, 24,200 mother-and-child sampling pairs were obtained from a total of 206,741 live births in Taiwan in 2005; 366 of the babies were born with the use of ART.

**Results:**

During pregnancy, mothers who used ART suffered from higher risks of complication than the natural conception counterparts, including gestational diabetes mellitus (GDM), pregnancy induced hypertension (PIH), and placenta previa. Additionally, babies born through ART had poorer outcomes than the natural conception groups: the low birth weight (<2500g) was 33.1% compared to 6.4% for babies born naturally.

**Conclusion:**

Pregnancy health and birth outcomes of women who underwent ART were worse than those who got natural conception. Types of maternal complication among ART women included GDM, PIH, and placenta previa. Having multiple births was the most important factor that causes low birth weight in babies. The results of this study can be used as a reference for the health and care of mothers and babies who use ART.

## Introduction

There is an upward trend for parents to resort to assisted reproductive technology (ART) treatment due to delayed childbirth or birth difficulties. According to statistics from the National Bureau of Health Promotion in Taiwan, there were 19,497 babies born through ART between 1998 and 2005 (1*).*

There are about 2% to 3% of babies born with the help of ART in European countries, and approximately 1% in the US (2, 3). With improvements in medical technology, changes are continually being made to ART and the success rate is improving. It has been well-known that births through ART increase the chances for maternal complications, multiple births, and the medical care required (3, 4). 

Negative outcomes such as multiple births, premature delivery, low birth weight (LBW), and small for gestational age (SGA) were also evidently numerous (3, 5). However, only few studies have investigated the influence of pregnancy health and lifestyle on babies born with low birth weight, and the sample size was also limited. 

The purpose of this study was to investigate the pregnancy health and lifestyle during pregnancy and postpartum of mothers who conceived by ART and how this influences birth outcomes, compared to those of natural conception through a national birth cohort study. The predictive factors of low birth weight babies<10 percentile of total sample norm who were born by ART were also examined.

## Materials and methods

**Population selection and sampling**

This study analyzed parts of the results of the first wave of the Taiwan Birth Cohort study. Taiwan Birth Cohort Study (TBCS) is a national population- based cohort study to detect the health status and its determinants of children in Taiwan. Sampled from Taiwan’s Birth Registration Database, there were 206,741 live births in 2005. 

A multi- stage stratified systematic sampling by district as well as urbanization was used in TBCS, and 24,200 pairs of mothers and their children were selected into the study (6), of which 21, 234 subjects (87.7%) completed the first wave survey (when the child was 6 months old) of TBCS. Written consents were provided by the mothers. The study was approved by the Medical Ethics Committee and Data Protection Board, National Department of Health, Taiwan. In this study, we divided all subjects (mother- child pairs) into two groups according to children born after ART and children born with naturally conceived pregnancies based on data collection through field interviews. 

**Data collection**

Trained research assistants collected data through structured questionnaires and in- depth interviews at subjects’ homes. The mothers were asked to answer questions regarding the type of conception [natural VS ART including intra cytoplasmic sperm injection (ICSI), gamete intrafallopian transfer (GIFT), zygote intra fallopian transfer (ZIFT) and in vitro fertilization (IVF)], demography, economic status, and history of any previous pregnancies and birth outcomes. 

The health information during pregnancy was obtained from the maternal health handbook (7), which recorded the results of 10 times prenatal checkup services provided by the National Health Insurance Program in Taiwan. The information on birth outcomes such as birth weight, body height, head circumference, congenital abnormalities, and method of delivery were collected from the birth certificate of each subject. 

**Statistical analysis**

SAS software was used to obtain descriptive and analytical statistics. Data were expressed as mean±SD and the rates were expressed as percentages. Differences in the pregnancy related complication, pregnancy outcome, and birth outcome between the ART and natural conception groups were determined using a chi-squared test or Wilcoxon two- sample test. 

The logistic regression model was used to estimate the effect of potential on whether or not inadequate birth weight (<10 percentile) of total sample norm showed any significance among babies born by ART.

## Results

Among the analyzed 21,234 live births in 2005 in Taiwan, 366 babies were born with the use of ART, making up 1.72% of the total live births. 

**Demographics and socioeconomic status of women who underwent ART treatment**

Mothers who used ART were usually older, with an average age of 33.2±4.1 years, while the average age of mothers who opted for natural conception was 28.8±4.9 years (p<0.001). The ART group received more education by finishing college (61.4%) than the natural conception group (45.8%). Fifty-seven point six percent (57.6%) of the ART group was employed (Table I). 

**Pregnancy health and lifestyle of women who underwent ART treatment**

The ART group mothers became pregnant at an average of 1.8 times (gravid), which was less than the natural conception group at 2.1 times (p<0.001). The numbers of spontaneous abortion and prenatal diagnosis in the ART group were higher than the natural conception group (p<0.001).

 The average weight of ART mothers before pregnancy was 55.1±9.0 kg, which was significantly higher than the average weight of natural conception mothers before pregnancy. The percentage of ART mothers who gained more than 12 kg during pregnancy was 67.5%, which was significantly higher than that of the natural conception group. 

ART group mothers suffered from a higher risk of pregnancy-related complications compared to the natural conception group counterparts, including gestational diabetes mellitus (GDM) (6.3% Vs. 2.2%), pregnancy induced hypertension (PIH) (4.1% Vs. 2%), and placenta previa (5.5% Vs. 1.7%). Fifty-one point nine percent (51.9%) of the ART group mothers were admitted during the pregnancy, which was significantly higher than their natural conception group counterparts (p<0.001) (Table II). 

Only 33.1% of births in the ART group were conducted through normal vaginal delivery (NVD) and 66.9% through caesarian section (C/S); while the rates of NVD and C/S in the natural conception group were 66.2% and 33.8%, respectively. A larger number (7.4%) of the ART group mothers experienced postpartum hemorrhage after childbirth than that (2.4%) of the natural conception group counterparts. About 16.9% of the ART group mothers claimed to suffer from postpartum depression (Table II).

The survey showed that ART group mothers smoked less than the natural conception group counterparts before pregnancy and during the first and second trimester of pregnancy. The ART group engaged in exercise more frequently than the natural conception group prior to the pregnancy, while the trend was reversed during pregnancy (Table III). 

**Perinatal outcome of babies born by ART**

The perinatal outcome of babies showed that 57.1% of babies conceived through ART were male and 42.9% were female, while the rates of naturally conceived male and female babies were 52.4% and 47.6%, respectively. The average birth weights were 2752±603g and 3110±443g in ART and natural conception group, respectively and low birth weights (<2500g) were 33.1% to 6.4%. Birth height was 47.8±3.6cm to 49.7±2.6cm and birth head circumference was 33.0±1.7cm to 33.3±1.7cm. 

The ART group mothers featured 55.7% single birth babies, 42.9% twins and 1.4% triplets, while the rates in the natural conception group were 98.1%, 1.9% and 0.03%, respectively. About 42.1% of babies conceived through ART were premature babies (<37 weeks). Disorders related to premature birth in babies born by ART were 9.6% as opposed to 4.9% in the natural conception group (Table IV). 

**Logistic regression analysis of inadequate birth weight (<10 percentile) among babies born by ART (** Table V **)**

Logistic regression analysis was conducted to investigate the associated factors to inadequate birth weight of ART-conceived babies. The odds ratio for inadequate birth weight among babies born by ART, multiple pregnancy was OR=26.28, for smoking prior to pregnancy OR=6.44, for having not complete 10 prenatal visit sessions OR=2.78, and for the mother’s weight before pregnancy OR=0.96.

**Table I:** Characteristics of mothers

		**ART group n=366**	**Non-ART group n=20868**	**p-value**
		**N (%)**	**N (%)**	
Age at delivery (years)		33.2±4.1	28.8±4.9	<0.001
Junior and senior high school		141 (38.6)	11202 (54.2)	<0.001
College and above occupation		224 (61.4)	9484 (45.8)	
Occupation				0.96
	No	155 (42.4)	8801 (42.2)	
	Yes	211 (57.6)	12047 (57.8)	

**Table II:** Comparison of obstetrics history between mothers of ART group and spontaneous group

		**ART group (n=366)**	**Non-ART group n=(20868)**	**RR**	**p-value**
		**N (%)**	**N (%)**		
Body weight before pregnancy (kg)		55.1± 9.0	53.2± 8.7		<0.001 a
Gravid		1.8± 1.0	2.1± 1.2		<0.001^a^
Spontaneous abortion					
	0	290 (79.2%)	18498 (88.6%)		<0.001^b^
	≧1	75 (20.8%)	2370 (11.4%)		
No. of antepartum examination					
	<10	72 (19.7%)	3614 (17.3%)	1.16 (0.90, 1.50) c	0.24
	≧10	294 (80.3%)	17254 (82.7%)		
Prenatal diagnosis					
	Yes	264 (72.5%)	12673 (61.0)	1.66 (1.33, 2.09) c	<0.001
	No	100 (27.5%)	8094 (39%)		
Body weight increased during pregnancy					
	≦12kg	119 (32.6%)	8204 (39.3%)		0.008 b
	>12kg	247 (67.5%)	12664 (60.7%)		
Hyperemesis gravid arum					
	Yes	94 (25.7%)	4949 (23.7%)	1.11 (0.88, 1.40) c	0.39
	No	272 (74.3%)	15899 (76.3%)		
Fever / infectious disease					
	Yes	17 (4.6%)	1353 (6.5%)	0.71 (0.44, 1.15) c	0.15
	No	349 (95.4%)	19485 (93.5%)		
Asthma					
	Yes	4 (1.1%)	175 (0.8%)	1.30 (0.49, 3.45) c	0.6
	No	362 (98.9%)	20672 (99.2%)		
Gestational diabetes Mellitus					
	Yes	23 (6.3%)	450 (2.2%)	2.95 (1.95, 4.45) c	<0.001
	No	342 (93.7%)	20388 (97.8%)		
Pregnancy induced hypertension					
	Yes	15 (4.1%)	412 (2%)	2.08 (1.25, 3.46) c	<0.001
	No	351 (95.9%)	20436 (98%)		
Premature rupture of membranes					
	Yes	61 (16.7%)	2805 (13.4%)	0.78 (0.59, 1.02) c	0.07
	No	305 (83.3%)	18058 (86.6%)		
Placenta previa					
	Yes	20 (5.5%)	353 (1.7%)	3.23 (0.20, 0.48) c	<0.001
	No	346 (94.5%)	20510 (98.3%)		
Abruption placenta					
	Yes	3 (0.8%)	87 (0.4%)	0.51 (0.17, 1.57) c	0.24
	No	363 (99.2%)	20776 (99.6%)		
Admitted during the pregnancy					
	Yes	190 (51.9%)	5092 (24.4%)		<0.001^b^
	No	176 (48.1%)	15771 (75.6%)		
Delivery					
	C/S	245 (66.9%)	7047 (33.8%)	3.86 (3.11, 4.79) c	<0.001
	NVD	121 (33.1%)	13818 (66.2%)		
Postpartum hemorrhage					
	Yes	27 (7.4%)	507 (2.4%)	3.26 (0.22, 0.48) c	<0.001
	No	339 (92.6%)	20356 (97.6%)		
Postpartum depression					
	Yes	62 (16.9%)	3605 (17.3%)	1.02 (0.78, 1.34) c	0.86
	No	304 (83.1%)	17246 (82.7%)		

a : Wilcoxon two-sample test.

b : Chi-Square test.

c : Relative risk.

**Table III:** Comparison of lifestyles between mothers of ART group and spontaneous group

		**ART group (n=366)**	**Non-ART group (n=20868)**	**p-value**
		**N (%)**	**N (%)**	
**Smoking**				
Prior to pregnancy				
	Yes	14 (3.8%)	1620 (7.8%)	0.005
	No	352 (96.2%)	19233 (92.2%)	
First trimester				
	Yes	5 (1.4%)	731 (3.5%)	0.03
	No	361 (98.6%)	20122 (96.5%)	
Second trimester				
	Yes	4 (1.1%)	595 (2.9%)	0.04
	No	362 (98.9%)	20258 (97.1%)	
**Drinking**				
Prior to pregnancy				
	Yes	39 (10.7%)	2363 (11.3%)	0.68
	No	327 (89.3%)	18485 (88.7%)	
First trimester				
	Yes	5 (1.4%)	340 (1.6%)	0.69
	No	361 (98.6%)	20509 (98.4%)	
Second trimester				
	Yes	31 (8.5%)	1960 (9.4%)	0.54
	No	335 (91.5%)	18886 (90.6%)	
**Exercise**				
Prior to pregnancy				
	Yes	128 (35.0%)	5509 (26.4%)	<0.01
	No	228 (65.0%)	15338 (73.6%)	
First trimester				
	Yes	104 (28.4%)	6863 (32.9%)	0.07
	No	262 (71.6%)	13958 (67.1%)	
Second trimester				
	Yes	56 (15.3%)	4056 (19.5%)	0.05
	No	310 (84.7%)	16781 (80.5%)	

Chi- Square test.

**Table IV:** Comparison of birth outcome between ART group and spontaneous group

		**ART group (N=366)**	**Non-ART group (N=20868)**	**RR**	**p-value**	
	
Gender (N%)					
	Male	209 (57.1%)	10936 (52.4%)	-	0.07
	Female	157 (42.9%)	9946 (47.6%)	-	
Body weight (Mean±SD)		2752±603	3110±443	-	<0.001^a^
	＜2500g (n%)	121 (33.1%)	1342 (6.4%)	-	<0.001^b^
	≧2500g (n%)	245 (66.9%)	19483 (93.6%)	-	
Body height(cm) (Mean±SD)		47.8±3.6	49.7±2.6	-	<0.001 a
Head circum(cm) (Mean±SD)		33.0±1.7	33.3±1.7	-	<0.001 a
Birth number (N%)					
	Singleton	204 (55.7%)	20471 (98.1%)	-	<0.001 b
	Twin	157 (42.9%)	390 (1.9%)		
	Triplet	5 (1.4%)	7 (0.03%)		
Premature (<37Wks) (N%)					
	Yes	154 (42.1%)	2249 (10.8%)	5.69 ( 4.65, 6.97) c	<0.001
	No	212 (57.9%)	18619 (89.2%)		
Disorders related to premature birth (N%)					
	Yes	35 (9.6%)	1014 (4.9%)	5.8 ( 3.27, 10.26) c	<0.001
	No	331 (90.4%)	19854 (95.1%)		

a : Wilcoxon two-sample test.

b : Chi-Square test.

c : Relative risk.

**Table V:** Logistic regression analysis of ART-conceived babies with low birth weight (<10 percentile) (N= 140/364).

**Variables**	**B**	**S.E.**	**OR**	**95%CI**	**p-value**
Body weight before pregnancy	-0.046	0.005	0.96	(0.95, 0.96)	<0.01
Less than 10 prenatal visit sessions	1.022	0.368	2.78	(1.36, 5.72)	0.006
Multiple birth	3.269	0.312	26.28	(14.24, 48.47)	<0.001
Smoking prior to pregnancy	1.862	0.749	6.44	(1.48, 27.92)	0.013

Adjusted variables: age, education, spontaneous abortion, pregnancy complications, Tocolysis, weight gain during pregnancy.

Nagel kerke R^2^: 58.1%.

B: Parameter estimate, S.E.: Standard error, OR: Odds ratio.

## Discussion

**Demographics and social exposure of women who underwent ART treatment**

The nationwide registry of ART conducted by the Bureau of Health Promotion in Taiwan between 2002 and 2005 showed that women who chose to undergo treatment rose from 32 to 34 years old (1). Lee *et al* in 1995 and 2010 (8, 9) reported that the average age of women who underwent ART was 32 years, the women suffered from 4 to 4.4 years of infertility, and they underwent between 2.5 and 3 years of treatment. This indicated that there is a substantial period between the discovery of the infertility problem and the actual treatment. The results of the present study showed that mothers who used ART were usually older, with an average age of 33.2±4.1 years, which was significantly higher compared to the average age of mothers who opted for natural conception, specifically 28.8±4.9 years (p<0.001). The reproductive capabilities of females decreased with an increase in age. Many factors influence the success rate of ART treatment and the age of the mother was viewed as the most important variable. 

The US CDC in 2002 demonstrated that live birth rate for 27 year old females who underwent treatment was 40%, while the rate went down to 6% for women at the age of 43; the number was further reduced to a level of 2% for women over the age of 43 (3). The mother’s age not only influenced the pregnancy success rate of ART treatment, but also increased the amount of maternal complications during pregnancy.

**Pregnancy health and lifestyle of women who underwent ART treatment**

Generally speaking, the ART group mothers had unfavorable pregnancy results. Although this group was less affected by fevers and other communicable diseases when compared to the natural conception group, it had a high percentage of GDM, PIH and placenta previa compared to the 

natural conception group, which was consistent with the results of previous studies (2, 10-13). In Romundstad *et al *nationwide population-based study in Norway (4), such variables as maternal age, parity, previous caesarean section, and time interval between pregnancies were controlled for showed that ART treatment increased the risk of placenta previa. Overall, the chances of maternal complications were higher for the ART treatment group than natural conception group. 

The current research showed that only 33.1% of mothers who underwent ART treatment gave birth through normal vaginal delivery while 66.9% chose the caesarian section. The percentage of normal vaginal delivery and caesarian section in the natural conception group was 66.2% and 33.8% respectively. Reubinoff *et al* (14) showed that for single birth babies, the percentage of caesarian section between IVF and natural conception groups was 41.9% and 15.5%, respectively. This indicated that a higher percentage of ART group mothers opted for caesarian section, and that this was not entirely influenced by multiple births. Approximately 7.4% suffered from postpartum hemorrhage, a number higher than that of the natural concept group mothers (2.4%). About 17% of ART- treated mothers confessed to suffering from postpartum depression, but this figure was not statistically significant when compared to the natural conception group. All these results were consistent with other series (15, 16). Smoking accelerates the coming of menopause, probably because it negatively affects the function of the ovary. Researches showed that smoking decreases the success rate of IVF by a level of 50% (17). The current survey showed that the percentage of ART group mothers who smoked before or during the first and second stages of pregnancy was lower than the natural conception group. The ART group engaged in exercise more frequently than the natural conception group prior to the pregnancy, while the trend was reversed during pregnancy. 

Furthermore, the current study showed that their living habits centered around healthy and unthreatening activities. Regarding smoking, a survey in Sweden found that women who underwent ART treatment smoked less than their natural conception (18). The present study resulted in the same conclusion.

**Perinatal outcome of babies born by ART**

The results of this study showed that babies born through ART treatment presented less desirable figures when compared to the naturally conceived group in terms of average birth weight, low birth weight (<2500g), height, and head circumference; they were also more at risk of being delivered prematurely and contracting diseases particular to premature babies. About 44.3% of births induced by ART were multiple births. A review of past literature showed that being pregnant with multiple births was one of the main reasons that complicated the birth conditions of ART treatment mothers (19). 

However, other studies reported that ART single birth babies made up a higher rate of death in newborns (OR 2.2), premature delivery (OR 2.0), low birth weight (OR 1.8), extremely low birth weight (OR 2.7), and SGA (OR 1.6) (2). In 2001, the ART monitoring project in Europe analyzed the data of 203,893 mothers who underwent ART treatment in 18 countries and found that multiple births made up 29.6% of IVF births, with twins making up 16.4% to 35.6% of the population, and triplets making up 0.4% to 11.9% of the total. In 2000, 35,000 babies were conceived with the help of ART treatment. Of these, 44% were twins, a figure that was 22 times more than under natural conception; and multiple births of 3 or more babies made up 9% of the total, a figure that was 50 times more than average (20). Being pregnant with multiple births caused mothers to have more complicated pregnancy and birth outcomes (19, 21).

**Logistic regression analysis of inadequate birth weight (<10 percentile) among babies born by ART**

Logistic regression analysis revealed that multiple births hiked up the odds ratio of babies conceived through ART treatment being born with a birth weight <10 percentile to as high as 26.28%. Multiple births associated with infertility treatment are recognized as an adverse outcome and are responsible for morbidities and mortality related to prematurity and low birth weight population. Reducing the number of embryos transferred and the use of natural cycle IVF were suggested by the Belgian project (22). 

Smoking could decrease the number of retrieved eggs and increase the time it takes to capture them during the ART cycle (23). Younglai, Holloway, and Foster’s meta-analysis showed that smoking decreases the success rate of conceiving through ART to approximately 50% (17). However, the effect of smoking on pregnancy outcomes in patients undergoing IVF is unclear. 

The present study showed that 3.8% of mothers in the ART group smoked before their pregnancy, and approximately 1.1 to 1.4% smoked during pregnancy. These figures were both lower than the average, and also lower than the figures found in Wright *et al*’s study (24). However, the results of this study showed that smoking hiked up the risk of birth weight of ART conceived babies to be in the <10 percentile. Thus, women undergoing ART cycles should be encouraged to stop smoking preferably before a cycle, although the time frame for the reversal of the adverse effect of smoking on ART outcomes is not clear (3).

Having not completed 10 prenatal visits elevated the risk of birth weight of ART conceived babies to be in the <10 percentile. The Department of Health in Taiwan provides pregnant women 10 free prenatal visits between conception and giving birth. The results of this study showed that not completing all ten sessions caused a higher probability of low birth weight, probably due to 52% were admitted into the hospital for prenatal care or prematurity.

The mother’s prenatal weight influenced the birth weight of ART-conceived babies to be in the <10 percentile of sample norms. Veleva, *et al* showed that obese and underweight women have an increased risk of miscarriage (25). Yeh, *et al* (26) also reported that based on the population-based study, both maternal pre-pregnancy BMI and total maternal weight gain are associated with increased odds ratio of twin newborn outcomes which will be very possible to cause low birth weight. We would like to suggest a continuous monitor on weight gain during pregnancy especially for women conceived by ART. 

Regarding the strengths of this study, the data in the current study was obtained from the national birth cohort (population-based study) with a relatively high response rate, and is thus fairly representative. However, as a national birth cohort study for multiple purposes, some data specific for ART, e.g. ART cycle and complications by the stage of pregnancy were not regularly collected. The pregnancy situation of women who underwent ART treatment was relatively less positive. Women considering ART treatment should be sufficiently informed of the complications involved in the treatment process. Multiple births are caused by a higher number of embryos injected during treatment to increase the success rate of each pregnancy; the practice is highly controversial. Better living habits during pregnancy and sufficient health care knowledge, combined with comprehensive prenatal care could improve the health of newborn babies.
